# The HSV-1 Exonuclease, UL12, Stimulates Recombination by a Single Strand Annealing Mechanism

**DOI:** 10.1371/journal.ppat.1002862

**Published:** 2012-08-09

**Authors:** April J. Schumacher, Kareem N. Mohni, Yinan Kan, Eric A. Hendrickson, Jeremy M. Stark, Sandra K. Weller

**Affiliations:** 1 Molecular, Microbial and Structural Biology Department, University of Connecticut Health Center, Farmington, Connecticut, United States of America; 2 Department of Biochemistry, Molecular Biology, and Biophysics, University of Minnesota Medical School, Minneapolis, Minnesota, United States of America; 3 Department of Cancer Biology, Beckman Research Institute of the City of Hope, Duarte, California, United States of America; University of Glasgow, United Kingdom

## Abstract

Production of concatemeric DNA is an essential step during HSV infection, as the packaging machinery must recognize longer-than-unit-length concatemers; however, the mechanism by which they are formed is poorly understood. Although it has been proposed that the viral genome circularizes and rolling circle replication leads to the formation of concatemers, several lines of evidence suggest that HSV DNA replication involves recombination-dependent replication reminiscent of bacteriophages λ and T4. Similar to λ, HSV-1 encodes a 5′-to-3′ exonuclease (UL12) and a single strand annealing protein [SSAP (ICP8)] that interact with each other and can perform strand exchange *in vitro*. By analogy with λ phage, HSV may utilize viral and/or cellular recombination proteins during DNA replication. At least four double strand break repair pathways are present in eukaryotic cells, and HSV-1 is known to manipulate several components of these pathways. Chromosomally integrated reporter assays were used to measure the repair of double strand breaks in HSV-infected cells. Single strand annealing (SSA) was increased in HSV-infected cells, while homologous recombination (HR), non-homologous end joining (NHEJ) and alternative non-homologous end joining (A-NHEJ) were decreased. The increase in SSA was abolished when cells were infected with a viral mutant lacking UL12. Moreover, expression of UL12 alone caused an increase in SSA, which was completely eliminated when a UL12 mutant lacking exonuclease activity was expressed. UL12-mediated stimulation of SSA was decreased in cells lacking the cellular SSAP, Rad52, and could be restored by coexpressing the viral SSAP, ICP8, indicating that an SSAP is also required. These results demonstrate that UL12 can specifically stimulate SSA and that either ICP8 or Rad52 can function as an SSAP. We suggest that SSA is the homology-mediated repair pathway utilized during HSV infection.

## Author Summary

The repair of DNA damage is essential to maintain genomic stability. Cells have at least four distinct DNA repair pathways, and defects in any of them can lead to tumor formation and cancer progression. Herpes Simplex Virus-1 (HSV-1) manipulates components of the host DNA repair pathways. In this paper we showed that DNA repair by the single strand annealing (SSA) pathway was increased during HSV infection and that other pathways were inhibited. We also show that a viral nuclease in conjunction with either a viral or cellular single strand annealing protein can stimulate the SSA pathway. We suggest that viral DNA synthesis occurs via an SSAdependent mechanism that is reminiscent of that used by bacterial viruses such as λ. Interestingly, λ has evolved an SSA-mediated repair mechanism to exchange genetic information that has also been used to enhance gene targeting in bacteria. It is thus possible that HSV proteins could be similarly used as tools to stimulate gene targeting in human cells leading to more effective strategies for gene therapy. Furthermore, the diversity of HSV reported in human populations, combined with the high rate of genetic exchange during infection, suggests that SSA may play a role in viral evolution and pathogenesis.

## Introduction

The 152 kb linear double-stranded DNA genome of Herpes Simplex Virus 1 (HSV-1) consists of unique components (U_L_ and U_S_) flanked by inverted repeat sequences. It has long been recognized that the U_L_ and U_S_ regions invert relative to one another during replication and that rates of recombination are high between co-infecting HSV viruses [1], [2], [3], [4], [5], [6]. For instance, recombination frequencies of 60% have been observed between HSV amplicons, and the rate of recombination between plasmids is higher in cells infected with HSV-1 than in uninfected cells or cells infected with SV40 [7], [8].

Despite the high level of recombination during infection little is known about the viral and cellular proteins involved, the mechanism of recombination or the importance of recombination during replication. HSV-1 encodes a 5′-to-3′ exonuclease, UL12, and a single stranded DNA binding protein with annealing activity (SSAP), ICP8. UL12 and ICP8 can perform a strand exchange reaction *in vitro* [9], [10] and are reminiscent of the complexes encoded by λ phage (Redα/β) and *Escherichia coli (E. coli)* (RecET), which can stimulate recombination-mediated genetic engineering (recombineering) [11], [12], [13], [14], [15], [16], [17]. In addition to providing a useful tool for genetic engineering, the λ recombination system plays an important role in the production of viral DNA concatemers necessary for encapsidation and the production of infectious progeny [18], [19]. Although it has been proposed that the HSV genome circularizes and rolling circle replication leads to the formation of concatemers, we and others have proposed that concatemer formation is more complex and may involve recombination-dependent replication reminiscent of bacteriophages λ and T4 [20].

The repair of DNA damage such as chromosomal double-strand breaks (DSBs), is essential to maintain chromosomal stability and prevent genetic loss. Eukaryotic cells have evolved at least four distinct repair pathways to repair DSBs, three which require some degree of homology [homologous recombination (HR), single strand annealing (SSA) and alternative non-homologous end joining (A-NHEJ)] and one that does not [classic non-homologous end joining (C-NHEJ)] [21], [22]. The two major homology-driven repair pathways, HR and SSA involve the formation of joint molecules by strand invasion (HR) or annealing (SSA). Strand invasion requires a RecA/Rad51 superfamily member while annealing is mediated by a single strand annealing protein (SSAP) [23], [24]. Mammalian RAD52 is an SSAP that promotes SSA, although RAD52 is not essential for SSA [25], [26]. The DSB repair pathways are activated through a complex series of signaling events that recognize and respond to DNA damage [27], [28]. Two of the major DNA damage sensing kinases, DNA-dependent protein kinase (DNA-PK) and ataxia-telangiectasia mutated (ATM), predominantly recognize DSBs [29], [30]. DNA-PK interacts with the Ku70/Ku80 heterodimer at DSBs and facilitates repair through C-NHEJ, while ATM is recruited to DSBs through an interaction with the MRN complex and facilitates homology-based repair [21], [28], [31], [32], [33]. Although it is known that HSV-1 manipulates components of the host double-strand break repair (DSBR) machinery during infection [34], [35], [36], [37], [38], [39], [40], [41], [42], [43], [44], [45], [46] it is not clear which repair pathways are active during infection. To address this question, we used chromosomally integrated reporter assays to measure the repair of DSBs by HR, SSA, A-NHEJ and C-NHEJ [26], [47], [48].

## Results

### HSV-1 infection increases SSA and decreases HR, A-NHEJ and NHEJ

**Figure 1:**
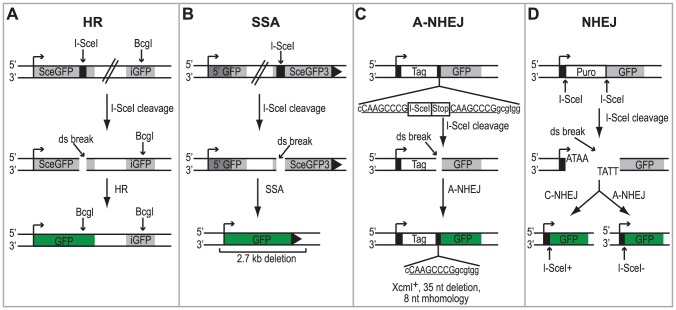
Schematic of the DSBR reporter assays. (A) DR-GFP reporter used to monitor HR [47]. SceGFP is a modified GFP gene, which contains an I-SceI site and in-frame termination codons. An 812-bp internal GFP fragment (iGFP) can repair the DSB by HR (gene conversion) and results in a functional GFP gene. (B) SA-GFP reporter used to monitor SSA [26]. 5′ GFP and SceGFP3 are GFP gene fragments which have 266 bp of homology (light gray). Repair of the DSB in SceGFP3 by SSA results in a functional GFP gene and a 2.7-kb deletion in the chromosome. Although an identical repair product could arise in this assay by HR with crossing over, this type of repair is estimated to be at least 30-fold less frequent [26]. (C) EJ2 reporter used to monitor A-NHEJ [48]. GFP is separated from an N-terminal tag (NLS/Zinc-finger) by an I-SceI site and stop codons in all three reading frames, which are flanked by 8 nts of microhomology. Repair of the DSB by A-NHEJ results in a functional GFP gene by restoring the coding frame between the tag and GFP as well as causing a 35 nt deletion. (D) EJ5 reporter used to monitor total NHEJ [48]. GFP is separated from a promoter by a puromycin gene flanked by two I-SceI sites. Excision of the puromycin gene and repair of the DSB by NHEJ joins the promoter with GFP thus creating a functional GFP gene. Two repair products can be formed, one that restores the I-SceI site (Ku-dependent C-NHEJ) and one that is I-SceI resistant (Ku-independent A-NHEJ).

Cell lines containing chromosomally integrated reporters have been developed to study the activation of the DSBR pathways following DNA damage [26], [47], [48]. In this study we utilized four HEK293 cell lines with chromosomally integrated reporters that monitor repair of an I-SceI generated DSB by HR, SSA, A-NHEJ or total NHEJ (both C-NHEJ and A-NHEJ) [48]. Each cell line contains a green fluorescent protein (GFP) reporter gene that is disrupted by the 18-bp recognition sequence for the I-SceI endonuclease and can no longer express GFP. Each reporter is designed in such a way that repair of the DSB using a specific DSBR pathway will restore the GFP reporter gene (see Figures 1A–1D for details). Therefore, repair can be measured in individual cells by monitoring the number of GFP-expressing cells using flow cytometry.

**Figure 2:**
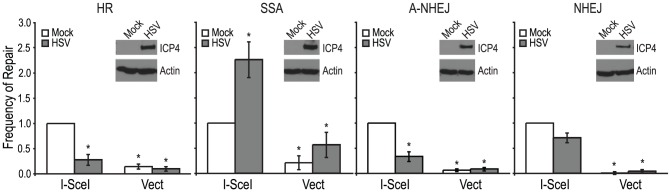
HSV infection increases SSA and inhibits HR, A-NHEJ and NHEJ. HR, SSA, A-NHEJ and NHEJ reporter cell lines were transfected with an empty vector or I-SceI expression vector and infected with HSV or mock infected. The average frequency of repair from at least four independent experiments, each performed with three independent samples were normalized to the I-SceI- transfected and mock infected samples. The HR and SSA experiments include the data from figures 3A, 4A and 4B as well as two additional experiments. Error bars represent the standard error of the mean. The asterisk indicates statistically significant differences from samples transfected with I-SceI and mock infected (P≤0.02). The insert shows a representative western blot analysis of cell lysates from mock- or HSV-infected cells. Infected cell lysates expressed the viral ICP4 protein and actin served as a loading control.

Each of the reporter cell lines were transfected with an I-SceI expression vector or an empty vector control. Four hours after transfection cells were infected with wild type HSV-1 (KOS) at a multiplicity of infection (MOI) of 2 PFU/cell and then analyzed by flow cytometry 36 hours post infection. Similar to previous reports, no significant GFP expression was detected in any of the cell lines transfected with the empty vector [26], [47], [48]. Interestingly, HSV infection caused a 2-fold increase in SSA over mock-infected cells; whereas, HSV infection resulted in decreased levels of HR, A-NHEJ and NHEJ (Fig. 2). Expression of the viral ICP4 protein confirmed that the cells were successfully infected (Fig. 2). These results indicate that a specific DSBR pathway is activated in HSV infected cells.

### The stimulation of SSA does not require viral DNA replication

**Figure 3:**
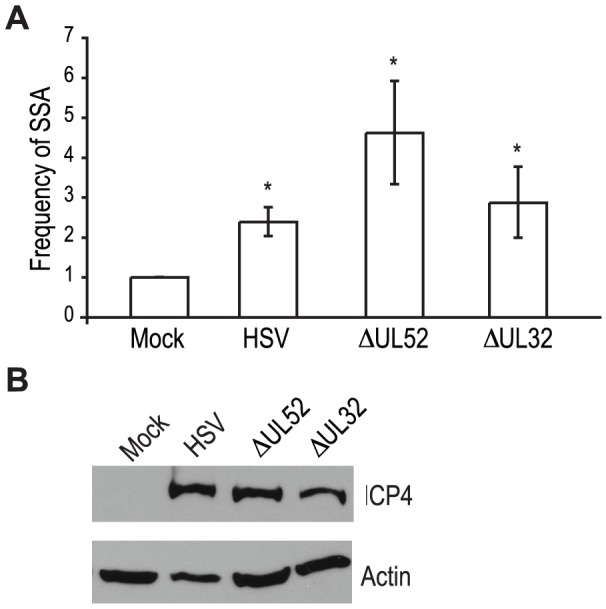
Viral DNA replication is not required to increase single strand annealing during HSV infection. (A) The SSA reporter cell line was transfected with an I-SceI expression vector and mock infected or infected with wild type HSV, or mutant viruses lacking UL52 (primase) or UL32 (packaging mutant). For each condition the average frequency of repair from three independent experiments, each performed with three independent samples were normalized to the I-SceI-transfected and mock-infected samples. Error bars represent the standard error of the mean. The asterisk indicates statistically significant differences from samples transfected with I-SceI and mock infected (P≤0.05). (B) Representative western blot analysis of cell lysates from mock or HSV, ΔUL52 or ΔUL32 infected cells. Infected cell lysates expressed the viral ICP4 protein and actin serves as a loading control.

Recombination is a frequent event during HSV-1 infection and it has been linked to viral DNA replication [1], [3], [20], [49]. In order to determine whether DNA replication is necessary for the increase in SSA, we analyzed cells infected with a DNA negative primase-null mutant (ΔUL52) and a DNA positive packaging-null mutant (ΔUL32) [50], [51] (Fig. 3A). Cells infected with ΔUL52 increased SSA approximately 4-fold over mock-infected cells and 2-fold over wild type-infected cells; whereas, ΔUL32 increased SSA approximately 3-fold, similar to wild type infected cells. Expression of the viral ICP4 protein confirmed the cells were infected (Fig. 3B). These results indicate that viral DNA replication is not necessary to increase the repair of a DSB in the cellular genome by SSA.

### UL12, but not ICP8 is necessary to increase SSA during HSV-1 infection

**Figure 4:**
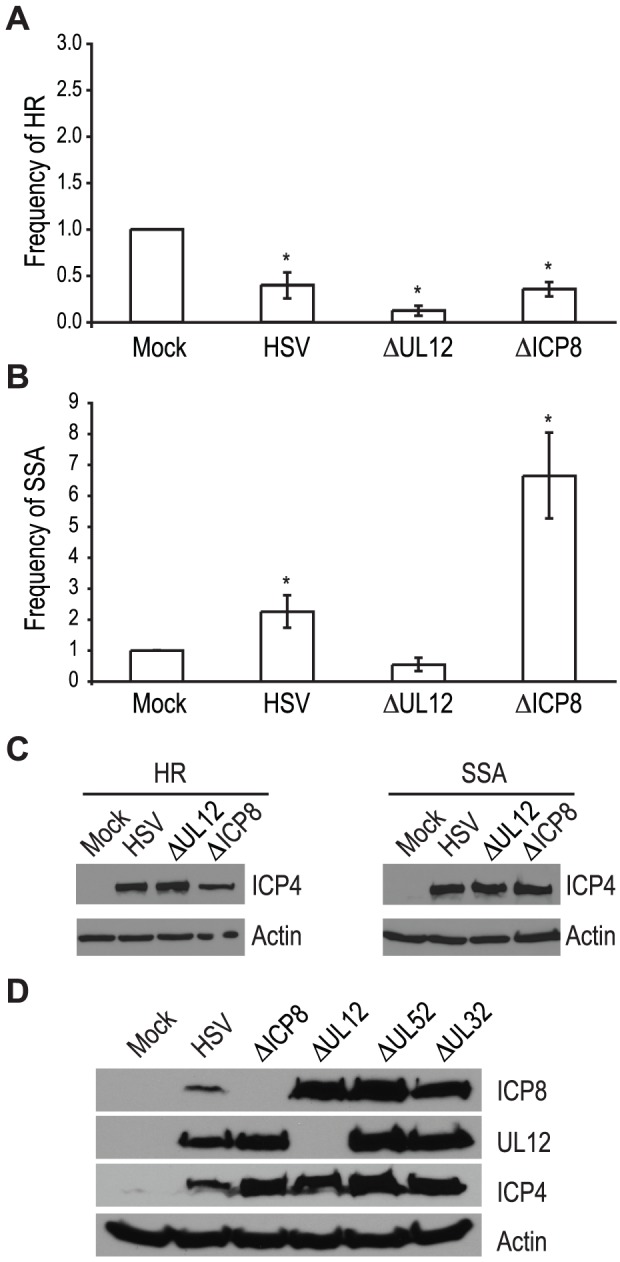
UL12 but not ICP8 is necessary to increase single strand annealing during HSV infection. (A) HR and (B) SSA reporter cell lines were transfected with an I-SceI expression vector and mock infected or infected with wild type HSV, or mutant viruses lacking UL12 or ICP8. The average frequency of repair for each condition was normalized as described in Figure 3. Error bars represent the standard error of the mean. The asterisk indicates statistically significant differences from samples transfected with I-SceI and mock infected (P≤0.01). (C) Representative western blot analyses of cell lysates from mock infected or HSV, ΔUL12 or ΔICP8 infected cells. Infected cell lysates expressed the viral ICP4 protein and actin serves as a loading control. (D) Representative western blot analysis of cell lysates from mock, HSV, ΔUL12, ΔICP8, ΔUL52 or ΔUL32 infected HEK293 cells. Cells were infected at a MOI of 2 PFU/cell and analyzed for expression 6 hours post infection.

The viral proteins UL12 and ICP8 form a two-component complex that is reminiscent of the λ Redα/β recombination system [9], [10]. Several lines of evidence suggest that Redα/β promotes recombination through an SSA mechanism [16], [17], [19], [52], [53], [54], [55], [56], [57]. Therefore, we next asked if UL12 and ICP8 are required for the increase in SSA and decrease in HR observed during infection. Infection with mutant viruses lacking UL12 and ICP8 (ΔUL12 and ΔICP8) [58], [59] caused decreases in HR similar to that observed during wild type HSV infection (Fig. 4A). On the other hand, the ΔUL12 virus completely eliminated the 2-fold increase in SSA observed during HSV infection (Fig. 4B). Expression of the viral ICP4 protein confirmed the cells were infected (Fig. 4C). These results indicate that UL12 is required to increase SSA during HSV infection.

The ΔICP8 virus increased SSA approximately 6-fold over mock-infected cells and 3-fold over wild type-infected cells (Fig. 4B). Since ICP8 is essential for HSV DNA replication and exhibits annealing activity *in vitro* [9], [10], [60], [61], we were surprised that a mutant virus lacking ICP8 exhibited a level of SSA greater than that seen in cells infected with wild type HSV. The lack of a specific requirement for ICP8 in SSA raises the possibility that a cellular annealing protein, such as Rad52 could substitute for ICP8 (discussed below).

The observation that both DNA negative viruses, ΔUL52 or ΔICP8, stimulated SSA levels to a greater extent than wild type HSV may indicate that UL12 levels are altered under conditions in which HSV DNA replication is inhibited. To rule out the possibility that increased SSA is due to elevated UL12 expression, we monitored ICP8 and UL12 expression in wild type or mutant-infected cells (Fig. 4D). In fact, UL12 levels were similar in all infected cells except those infected with ΔUL12. ICP8 expression levels were similar in cells infected with the UL12, UL52 and UL32 deficient viruses, but were elevated over ICP8 expression in wild type-infected cells. In addition, the expression levels of several SSA proteins (*e.g.*, Rad52, MSH2 and MSH3) are stable during infection [35], [41]. Thus, the expression levels of viral and cellular proteins cannot account for the enhanced SSA levels observed for replication incompetent HSV.

It is also possible that UL12 activity is deregulated in the absence of HSV DNA replication. Deregulated nuclease activity may lead to more extensive resection and increased single strand annealing. UL12 is known to interact with ICP8, MRN and two mismatch repair (MMR) complexes, MSH2-MSH6 and MSH2-MSH3 [35], [62], [63]. In the absence of viral DNA replication, the reorganization of cellular and viral proteins to replication compartments does not occur. Thus, UL12 and other viral and cellular repair proteins may be more available to interact with cellular DNA. Another possibility is that UL12 is regulated by post-translational modifications. UL12 is phosphorylated on at least five residues ([64]; Balasubramanian and Weller, unpublished), and some of these phosphorylation events may be altered in the absence of DNA synthesis.

### Expression of UL12 is sufficient to increase SSA and requires its 5′-to-3′ exonuclease activity

**Figure 5:**
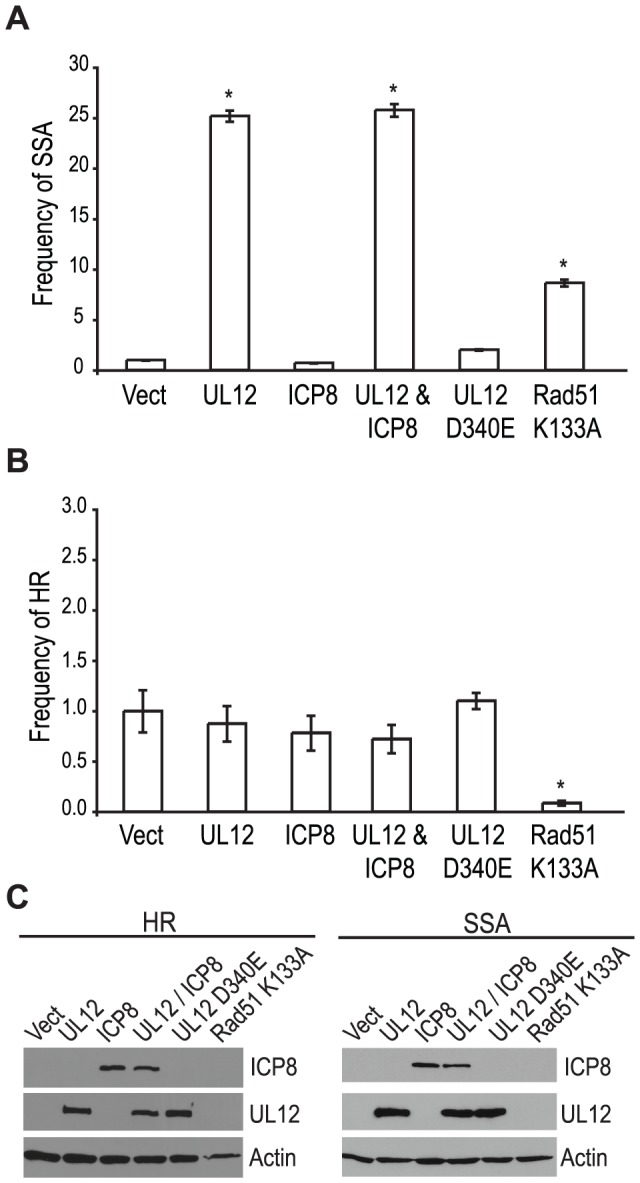
UL12 is sufficient to increase single strand annealing and is dependent on its exonuclease activity. (A) SSA and (B) HR reporter cell lines were transfected with the I-SceI expression plasmid and plasmids expressing UL12, ICP8, UL12 D340E or Rad51 K133A as indicated. For each condition the average frequency of repair from at least three independent experiments, each performed with three independent samples were normalized to the I-SceI and empty vector transfected sample. Error bars represent the standard error of the mean. The asterisk indicates statistically significant differences from samples transfected with I-SceI and an empty vector (P≤0.007 except for the SSA reporter cell line expressing Rad51 K133A where P = 0.01). (C) Representative western blot of cell lysates transfected with an empty vector or vectors expressing ICP8, UL12, UL12 D340E or Rad51 K133A. Actin serves as a loading control.

Since a mutant virus lacking UL12 was unable to increase SSA we asked if expression of UL12 alone was sufficient. The SSA reporter cells were transfected with the I-SceI expression plasmid and plasmids expressing UL12, ICP8, UL12 D340E, or Rad51 K133A and analyzed for GFP expression approximately 72 hours post transfection. Rad51 K133A is a dominant negative mutant of Rad51 that was previously demonstrated to reduce HR and increase SSA and was used as a control for increased SSA activity [26], [65]. Impressively, UL12 expression caused a 25-fold increase in SSA while the expression of Rad51 K133A only resulted in a 8-fold increase (Fig. 5A). Stimulation of SSA by UL12 was completely eliminated in cells transfected with a plasmid expressing a catalytically dead UL12 mutant (UL12 D340E [66]) (Fig. 5A). Coexpression of UL12 and ICP8 did not alter the UL12 mediated increase in SSA (Fig. 5A) consistent with earlier results (Fig. 4B) suggesting that ICP8 is not a key SSA regulatory factor. UL12 and ICP8 expression was confirmed by western blot (Fig. 5C). Expression of Rad51 K133A was inferred from its activity in the SSA assay as described previously [26], [65]. A similar increase in SSA (approximately 20-fold) was observed when UL12 or UL12 and ICP8 were expressed in U20S cells containing the SSA reporter (data not shown).

In order to determine if the effect of UL12 is specific for SSA we asked if expression of UL12 and/or ICP8 could affect the frequency of HR. Consistent with previously published reports, expression of the Rad51 K133A mutant resulted in a 12.5-fold decrease in HR (Fig. 5B) [26], [65]; whereas, expression of UL12 in the presence or absence of ICP8 had no effect on HR. UL12 and ICP8 expression was confirmed by western blot (Fig. 5C). These results demonstrated that UL12 is necessary and sufficient to specifically increase SSA and that the 5′-to-3′ exonuclease activity of UL12 is required.

### The UL12-mediated increase in SSA requires a cellular or viral SSAP

**Figure 6:**
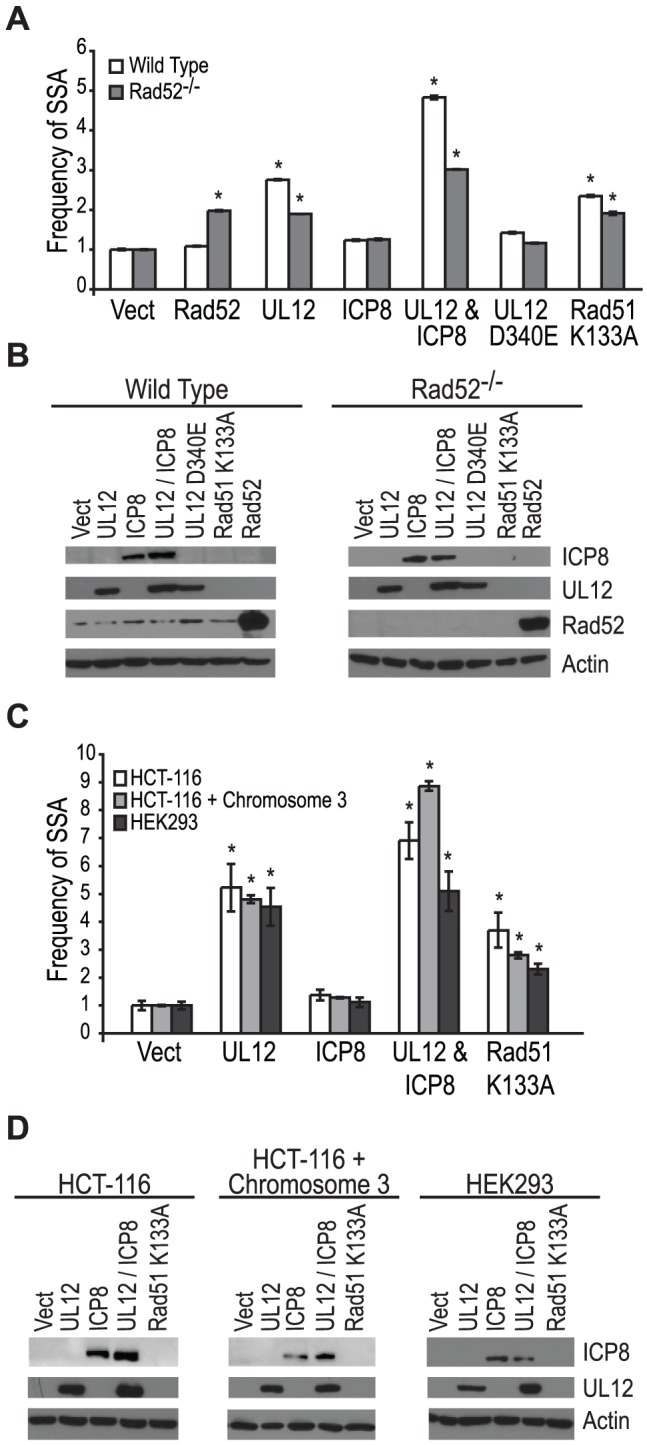
Rad52 and/or ICP8 are necessary for the UL12-mediated increase in SSA. (A) SSA reporter cell lines were transfected with the I-SceI expression plasmid and plasmids expressing UL12, ICP8, UL12 D340E, Rad51 K133A or mRad52 as indicated. For each condition the average frequency of repair from at least three independent experiments, each performed with two or three independent samples were normalized to the I-SceI and empty vector transfected sample. Error bars represent the standard error of the mean. The asterisk indicates statistically significant differences from samples transfected with I-SceI and an empty vector (P≤0.004). (B) Representative western blot of cell lysates transfected with an empty vector or vectors expressing ICP8, UL12, UL12 D340E, Rad51 K133A or mRad52. Actin serves as a loading control. (C) HCT-116, HCT-116+chromosome 3 and HEK293 cell lines were transfected with a plasmid based SSA reporter, I-SceI expression plasmid and plasmids expressing UL12, ICP8, or Rad51 K133A as indicated. For each condition the average frequency of repair from at least two independent experiments, each performed with two independent samples were normalized to the I-SceI and empty vector transfected sample. Error bars represent the standard error of the mean. The asterisk indicates statistically significant differences from samples transfected with I-SceI and an empty vector (P≤0.03). (D) Representative western blot of cell lysates transfected with an empty vector or vectors expressing ICP8, UL12, or Rad51 K133A. Actin serves as a loading control.

Coexpression of ICP8 with UL12 had no effect on SSA, and cells infected with the ΔICP8 virus caused an increase in SSA greater than that seen during wild type infection (Fig. 4B and Fig. 5A). At face value, these data suggested that ICP8 expression was not required for SSA and, by inference, further suggested that there must be a host factor capable of functionally substituting for it. We therefore asked if the cellular annealing protein Rad52 could substitute for ICP8 by integrating the SSA reporter plasmid into wild type and Rad52 null (Rad52^−/−^) derivatives of the human colorectal carcinoma cell line, HCT-116 (Kan *et al.* manuscript in preparation). Wild type and Rad52^−/−^ cells were transfected with the I-SceI expression plasmid, and plasmids expressing UL12, ICP8, UL12 D340E, Rad51 K133A, or mouse Rad52 (mRad52) and analyzed for GFP expression approximately 72 hours post transfection. Expression of mRad52 had no significant effect on SSA in the wild type cells and caused a 2-fold increase in the Rad52^−/−^ cells as compared to Rad52^−/−^ cells transfected with an empty vector. This result is consistent with previously published reports in Rad52^−/−^ murine embryonic stem cells [26] and indicates that Rad52 promotes SSA. In cells lacking Rad52 the UL12-mediated stimulation of SSA was decreased as compared to wild type cells (Fig. 6A). Coexpression of ICP8 and UL12 in the Rad52^−/−^ cells caused a 3-fold increase in SSA similar to UL12 expression alone in wild type cells. Interestingly, coexpression of UL12 and ICP8 in wild type HCT-116 cells increased SSA approximately 5-fold over the vector control and 2-fold over cells expressing UL12 alone (Fig. 6A). UL12, ICP8, and Rad52 expression was confirmed by western blot (Fig. 6B). These results indicate that either Rad52 or ICP8 can promote the UL12-mediated increase in SSA, and that coexpression of ICP8 and UL12 can further increase SSA in HCT-116 cells. In contrast, expression of UL12 in U20S or HEK293 cells resulted in a 20- or 25-fold increase in SSA that was not further elevated by coexpression of ICP8 (Fig. 5 and data not shown). This cell type difference may indicate that SSAP activity is not limiting for SSA in HEK293 cells. These results demonstrate that either Rad52 or ICP8 are necessary for the UL12-mediated increase in SSA and that in some cell types coexpression of ICP8 and UL12 can further increase SSA.

HCT-116 cells do not express MLH1 and are defective for MMR [67]. In order to determine if the different effect of UL12 and ICP8 on SSA in HEK293 and HCT-116 cells was due to the loss of MMR in HCT-116 cells we used the complemented cell line HCT-116+chromosome 3 in which MLH1 expression has been restored [68]. Each cell line was transfected with a plasmid based version of the SSA reporter, the I-SceI expression plasmid and plasmids expressing UL12, ICP8, or Rad51 K133A and analyzed for GFP expression approximately 48 hours post transfection. UL12 expression increased SSA approximately 5-fold in all three cell lines (Fig. 6C). Coexpression of UL12 and ICP8 in HEK293 cells increased SSA approximately 5-fold similar to expression of UL12 alone (Fig. 6C). In contrast, coexpression of UL12 and ICP8 increased SSA approximately 7- and 9-fold in HCT-116 and HCT-116+chromosome 3 cells, respectively (Fig. 6 C). UL12 and ICP8 expression was confirmed by western blot (Fig. 6D). Expression of MHL1 in the complemented cell line has been shown previously (data not shown and [35]). These results demonstrate that UL12 alone is sufficient to increase SSA in all three cell lines and that coexpression of ICP8 and UL12 can further enhance SSA in HCT-116 and HCT-116+chromosome 3 cells, but not HEK293 cells. Thus, while there is a cell type difference between HEK293 and HCT-116 cells, this difference is not due to the loss of MMR.

## Discussion

Several lines of evidence indicate that HSV-1 manipulates components of the DSBR pathways [34], [35], [36], [37], [38], [39], [40], [41], [42], [43], [44], [45], [46]; however, the question of which DSBR pathways are functional during infection had never been addressed. Reporter assays developed to distinguish between DSBR pathways demonstrated that repair by SSA was increased in HSV-infected cells, while repair by HR, NHEJ or A-NHEJ was decreased. Infection with a viral mutant lacking UL12 abolished the increase in SSA observed during wild type infection. Furthermore, expression of UL12 alone caused an increase in SSA, which was dependent on its exonuclease activity. An SSAP was also required as UL12-mediated increases in SSA were decreased in cells lacking the cellular SSAP, Rad52, and could be restored by coexpressing the viral SSAP, ICP8. Coexpression of UL12 and ICP8 increased SSA greater than UL12 alone in HCT-116 cells. Thus, we have demonstrated that in HSV-infected cells a specific DSBR pathway (SSA) becomes activated.

Of the four DSBR pathways, C-NHEJ is the only one that does not involve resection and some degree of homology. During HSV infection ICP0 causes the degradation of DNA-PKcs (a key C-NHEJ factor) in some but not all cell types [37], [41], [44], Furthermore, viral yields are increased in cells deficient for DNA-PKcs or Ku70 [37], [39], [44]. Thus it appears that DNA-PKcs and Ku70 exert a negative effect on viral infection; however, knockdown of DNA Ligase IV or XRCC4 decreased viral yields and suggests that some components of the C-NHEJ pathway may be important for viral replication [46]. The observation that NHEJ is decreased in cells infected with HSV is consistent with the prediction that degradation of DNA-PKcs would lead to inactivation of NHEJ. It will be of interest to determine whether this decrease can be attributed to ICP0 or if other viral proteins are involved.

In contrast, components of the ATM-mediated DNA damage response are recruited to viral replication compartments (ATM, MRN, RPA, MSH2-MSH6, BRCA1, BLM, WRN and RAD51), and several exert a beneficial effect on HSV infection [35], [36], [37], [38], [39], [40], [41], [43], [44], [45]. Since ATM activation can result in the repair of DSBs by the major homology driven pathways, HR and SSA [69], the data imply that one (or both) of these pathways may be beneficial for HSV. There is precedent for this: λ can utilize either Redα or RecA to mediate recombination by SSA or HR [57], [70], [71], [72], [73], [74], [75] and human cytomegalovirus (HCMV) has recently been reported to increase HR during infection by a mechanism that is mediated by the immediate early protein IE1–72 [76]. In this study we report that HSV infection decreased the levels of HR while increasing the levels of SSA. Although our data suggests that SSA may be the pathway of choice for recombination between viral genomes, it should be noted that the assays used in this study monitor events occurring on cellular chromatin. It is possible that the decrease in HR seen during HSV infection is due to the sequestration of HR proteins away from the cellular DNA. To conclusively rule out a role for HR, it will be necessary to employ a viral genome based recombination assay under conditions in which either UL12, ICP8, or both, are lacking.

UL12 interacts specifically with the MRN complex (comprised of Mre11, Rad50 and Nbs1) [62]. MRN recruits CtIP and other end resection proteins that are essential for promoting homology-mediated repair [21], [77], [78], [79]. It is possible that the UL12∶MRN interaction influences the probability that resection will occur at a DSB as well as the recruitment of other repair factors ultimately affecting the mechanism of repair. UL12 also interacts with two mismatch repair (MMR) complexes, MSH2–MSH6 and MSH2–MSH3 [35]. MMR proteins play roles in preventing recombination between heterologous sequences as well as facilitating flap removal during SSA [80], [81], [82]. In this study, we demonstrated that UL12 is necessary and sufficient to increase SSA and that the 5′-to-3′ exonuclease activity of UL12 is required. Thus, we speculate that the interactions between UL12 and cellular repair proteins play a role in shifting DSBR toward the SSA pathway.

**Figure 7:**
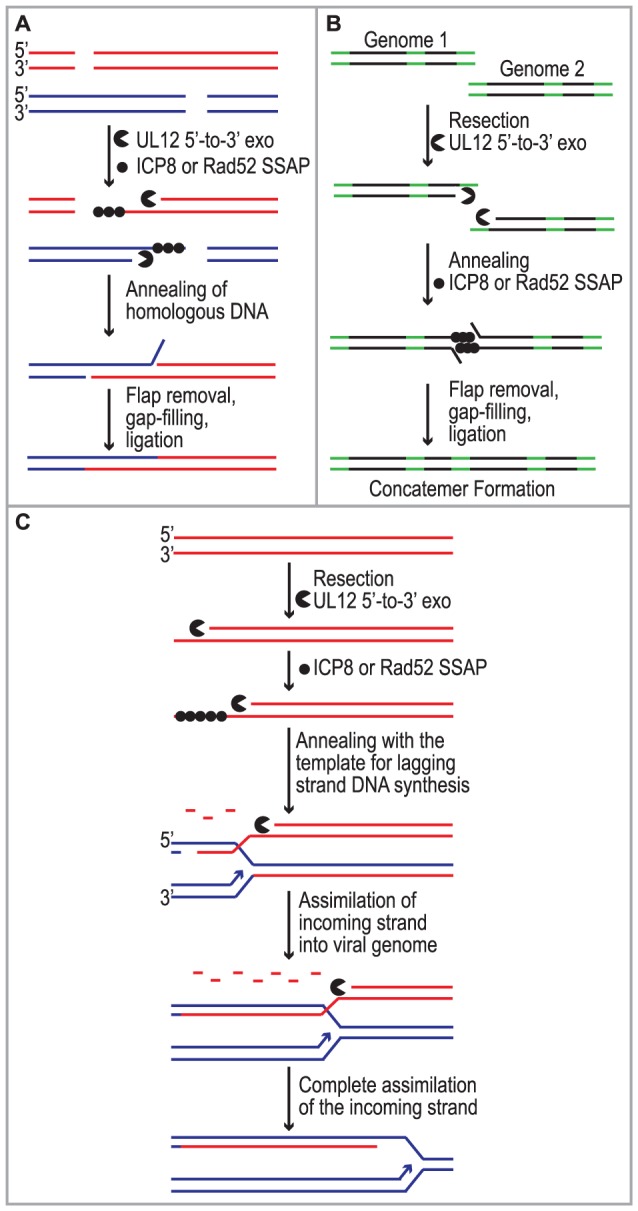
Implications for single strand annealing during HSV infection. Three models depict potential roles for SSA during HSV infection. (A) SSA is used to repair DSBs that arise as a consequence of DNA replication. (B) Resection and annealing of the repeat regions (green) at viral termini leads to concatemer formation. (C) SSA-mediated replication-dependent recombination occurs similar to the new recombination model proposed for λ phage.

Despite studies identifying HSV *cis*- and *trans*-acting factors that are required for HSV replication and circumstantial evidence that host proteins may be involved, the mechanism of concatemer formation during HSV infection is not understood. It has long been recognized that recombination is a frequent event during HSV-1 infection and may be responsible for the isomerization of the viral genome as well as concatemer formation [1], [2], [3], [4]. Recombination could also play a role in DNA replication reminiscent of recombination-dependent replication used by bacteriophage. The observations made in this study are consistent with several possible roles for SSA during HSV infection (Fig. 7). We and others have proposed that DSBs arise during HSV infection and that they may stimulate homologous recombination (reviewed in [20]). Based on the finding that SSA is stimulated in HSV-infected cells we suggest that SSA could be used to repair DSBs that arise as a consequence of DNA replication through nicks and gaps known to exist in viral genomes [20], [83], [84]. Under this scenario, recombinant molecules could arise as a result of resection by UL12 and annealing of homologous regions by Rad52 and/or ICP8 (Fig. 7A). SSA may also be utilized during early stages of infection for the formation of circular or concatemeric DNA. For instance, if the ends of the viral genome are not protected, resection and annealing of the repeat regions at viral termini could lead to genome circularization or concatemer formation (for simplicity, only concatemers are shown in Fig. 7B). These models, however, do not fully explain the apparent link between recombination and replication in cells infected with HSV. Complex branched structures are formed during viral DNA replication, and genomic inversions occur at the earliest times that replicated DNA can be detected and are mediated by the viral replication machinery [5], [49], [85], [86], [87]. These observations are not consistent with a simple rolling circle model for HSV DNA replication, and we have suggested that by analogy with the bacteriophages λ and T4, recombination may play a role during viral DNA synthesis [20].

Previous models suggested that the function of λ Redα is to carry out limited resection at a DSB or end resulting in a double-stranded region flanked by a 3′ overhang, which could lead to recombination through strand invasion (HR), strand annealing (SSA) or replisome invasion [13], [17], [53], [55], [56], [57], [88]. A recent set of experiments from the Church and Stewart laboratories, however, supports a new model for SSA-mediated replication-dependent recombination [89], [90]. These authors suggest that Redα functions to completely degrade one entire strand of a duplex molecule leaving the other intact. The remaining single strand is then incorporated into a replication fork by annealing to the lagging strand template in a reaction dependent on the SSAP (Redβ). One very appealing aspect to this model is that it explains the link between recombination and DNA replication. It has long been observed that efficient DNA recombination between λ chromosomes relies on DNA synthesis and that the λ recombination system plays an important role in the production of viral DNA concatemers necessary for encapsidation and the production of infectious progeny [18], [19].

We reported that similar to λ Redα/β, HSV UL12/ICP8 can mediate strand exchange *in vitro* and that UL12 shares several conserved sequence motifs with Redα [9], [10], [66], [91]. Furthermore, we now report that expression of UL12 caused an increase in levels of SSA that can be further elevated by coexpression of ICP8 in some cell types. We thus propose that UL12 and an SSAP (ICP8 and/or Rad52) mediate double-stranded DNA recombination similar to that proposed for λ (Fig. 7C). It is thus possible that SSA-mediated recombination plays an important role in the HSV life cycle not only to generate concatemers suitable for encapsidation but also to restart DNA replication at a stalled replication fork. This mechanism might also facilitate the generation of complex branched molecules seen in replication intermediates during HSV infection and provides a viable alternative to the rolling circle replication mechanism. Additional experiments will be necessary to determine the actual role of SSA during HSV infection.

The Redα/β system has also been used extensively as a tool for genetic engineering and gene targeting [11], [12], [13], [14], [15], [16]. For instance, expression of Redα/β can stimulate recombination-mediated genetic engineering or “recombineering” to construct targeted gene deletions, insertions or point mutations [11], [12], [16], [92], [93], [94]. Taken together these observations raise the interesting possibility that UL12/ICP8 could be used in mammalian cells to stimulate recombination-mediated genetic engineering leading to more effective strategies for gene therapy. Extensive recombination between viral genomes also provides a mechanism to promote genetic variation [95]. The diversity observed in human populations of VZV, HSV and HCMV combined with the high rates of recombination in herpesviruses suggests that recombination may play a role in herpesvirus evolution and pathogenesis [96], [97], [98], [99] and thus clearly warrants additional investigation.

## Materials and Methods

### Viruses

The HSV-1 strain KOS was used as the wild type strain in all experiments. The 5′-to-3′ exonuclease null virus, AN-1, contains an ICP6:lacZ insertion in the UL12 gene [59]. The ICP8 (HSV-1 SSAP) null virus, HD2, contains an in-frame lacZ insertion mutation in the UL29 gene and was provided by David Knipe (Harvard Medical School, Cambridge MA) [58]. The primase-null virus, *hr114*, contains an ICP6::lacZ insertion mutation in the UL52 gene [50]. The packaging-null mutant, *hr*64, contains an ICP6::lacZ insertion mutation in the UL32 gene [51]. All mutant viruses were constructed using a KOS background.

### Cell lines and plasmids

The HEK293 and HCT-116 cell lines were obtained from the American Type Culture Collection (ATCC). The HCT-116+chromosome 3 cells were kindly provided by Thomas Kunkel and Alan Clark [68]. The HEK293 SA-GFP, DR-GFP, EJ5-GFP and EJ2-GFP cell lines were described previously [48]. The HCT-116 Rad52^−/−^ cell line (Kan *et al.*, manuscript in preparation) was generated by rAAV gene targeting technology as previously described [100]. Stable integrants of the SSA reporter were generated by transfecting wild type HCT-116 and Rad52^−/−^ cell lines with the SA-GFP reporter plasmid [26] by Lipofectamine 2000-mediated transfection (Invitrogen). Puromycin was added 24 hours later at 2 µg/ml. Puromycin resistant colonies were screened for a functional reporter by transfecting an empty vector or the I-SceI and Rad52 expression plasmids. 48 to 72 hours post transfection cells were analyzed by flow cytometry for GFP expression. The empty vector, pSAK, and I-SceI, Rad51-K133A, UL12, UL12 D340E, mouse Rad52 and Cherry expression vectors have been described [65], [66], [101], [102], [103], [104]. The ICP8 expression vector was created by PCR amplifying ICP8 from pCM-DBP and cloned using HindIII/EcoRI into pSAK [102], [105].

### Western blots

Cell lysates for the DNA repair assays were obtained by pooling duplicate samples following analysis by flow cytometry. Cells were pelleted and lysed in 2X SDS sample buffer [35]. HSV HEK293 infected cell lysates were obtained as described previously [35]. Cell lysates were run on 10% SDS-Page gels, transferred to PVDF and immunoblotted with antibodies to ICP4 (US Biological), ICP8 (Abcam ab20194), Rad52 (Santa Cruz) UL12 [gift from Joel Bronstein and Peter Weber (Parke-Davis Pharmaceutical)] and Actin (Sigma) as described previously [35].

### Repair assays

The repair of I-SceI-generated dsDNA breaks has been described previously [26], [47], [48]. Briefly, 2×10^5^ HEK293 DR-GFP, SA-GFP, EJ5-GFP, EJ2-GFP or 3×10^5^ wild type or Rad52^−/−^ HCT-116 SA-GFP cells were plated in 12 well plates. The next day, cells were transfected with various combinations of plasmids expressing I-SceI, Cherry, UL12, UL12 D340E, ICP8, Rad51-K133A or mouse Rad52 by Lipofectamine 2000-mediated transfection as recommended by the manufacturer (Invitrogen). For each experiment an equivalent amount of empty vector (pSAK) was included in parallel transfections. Approximately 72 hours post transfection cells were analyzed by flow-cytometry for GFP and Cherry expression. Cherry expression served as a transfection control. Plasmid based repair assays were done as described above except cells were also transfected with the SSA reporter plasmid SA-GFP [26]. Approximately 48 hours post transfection cells were analyzed by flow cytometry for GFP and Cherry expression. Repair assays done during HSV infection were transfected as described above. Four hours post transfection the media was removed and the cells were infected with HSV-1 at a MOI of 2 PFU/cell as described previously [106]. Following infection the cells were moved to 34°C. Cells were fixed with 4% paraformadehyde and analyzed by flow cytometry for GFP and Cherry expression 36 hours post infection.

## References

[1] Brown SM, Ritchie DA, Subak-Sharpe JH (1973) Genetic studies with herpes simplex virus type 1. The isolation of temperature-sensitive mutants, their arrangement into complementation groups and recombination analysis leading to a linkage map. J Gen Virol 18: 329–346. 4348796 10.1099/0022-1317-18-3-329

[2] Hayward GS, Jacob RJ, Wadsworth SC, Roizman B (1975) Anatomy of herpes simplex virus DNA: evidence for four populations of molecules that differ in the relative orientations of their long and short components. Proc Natl Acad Sci U S A 72: 4243–4247. 172900 10.1073/pnas.72.11.4243 PMC388696

[3] Schaffer PA, Tevethia MJ, Benyesh-Melnick M (1974) Recombination between temperature-sensitive mutants of herpes simplex virus type 1. Virology 58: 219–228. 4362547 10.1016/0042-6822(74)90156-1

[4] Sheldrick P, Berthelot N (1975) Inverted repetitions in the chromosome of herpes simplex virus. Cold Spring Harb Symp Quant Biol 39 Pt 2: 667–678. 169022 10.1101/sqb.1974.039.01.080

[5] Zhang X, Efstathiou S, Simmons A (1994) Identification of novel herpes simplex virus replicative intermediates by field inversion gel electrophoresis: implications for viral DNA amplification strategies. Virology 202: 530–539. 8030219 10.1006/viro.1994.1375

[6] Delius H, Clements JB (1976) A partial denaturation map of herpes simplex virus type 1 DNA: evidence for inversions of the unique DNA regions. J Gen Virol 33: 125–133. 185323 10.1099/0022-1317-33-1-125

[7] Dutch RE, Bianchi V, Lehman IR (1995) Herpes simplex virus type 1 DNA replication is specifically required for high-frequency homologous recombination between repeated sequences. J Virol 69: 3084–3089. 7707536 10.1128/jvi.69.5.3084-3089.1995 PMC189009

[8] Fu X, Wang H, Zhang X (2002) High-frequency intermolecular homologous recombination during herpes simplex virus-mediated plasmid DNA replication. J Virol 76: 5866–5874. 12021319 10.1128/JVI.76.12.5866-5874.2002 PMC136225

[9] Reuven NB, Staire AE, Myers RS, Weller SK (2003) The herpes simplex virus type 1 alkaline nuclease and single-stranded DNA binding protein mediate strand exchange in vitro. J Virol 77: 7425–7433. 12805441 10.1128/JVI.77.13.7425-7433.2003 PMC164775

[10] Reuven NB, Willcox S, Griffith JD, Weller SK (2004) Catalysis of strand exchange by the HSV-1 UL12 and ICP8 proteins: potent ICP8 recombinase activity is revealed upon resection of dsDNA substrate by nuclease. J Mol Biol 342: 57–71. 15313607 10.1016/j.jmb.2004.07.012 PMC4412345

[11] Ellis HM, Yu D, DiTizio T, Court DL (2001) High efficiency mutagenesis, repair, and engineering of chromosomal DNA using single-stranded oligonucleotides. Proc Natl Acad Sci U S A 98: 6742–6746. 11381128 10.1073/pnas.121164898 PMC34423

[12] Murphy KC (1998) Use of bacteriophage lambda recombination functions to promote gene replacement in Escherichia coli. J Bacteriol 180: 2063–2071. 9555887 10.1128/jb.180.8.2063-2071.1998 PMC107131

[13] Court DL, Sawitzke JA, Thomason LC (2002) Genetic engineering using homologous recombination. Annu Rev Genet 36: 361–388. 12429697 10.1146/annurev.genet.36.061102.093104

[14] Muyrers JP, Zhang Y, Testa G, Stewart AF (1999) Rapid modification of bacterial artificial chromosomes by ET-recombination. Nucleic Acids Res 27: 1555–1557. 10037821 10.1093/nar/27.6.1555 PMC148353

[15] Zhang Y, Buchholz F, Muyrers JP, Stewart AF (1998) A new logic for DNA engineering using recombination in Escherichia coli. Nat Genet 20: 123–128. 9771703 10.1038/2417

[16] Yu D, Ellis HM, Lee EC, Jenkins NA, Copeland NG, et al (2000) An efficient recombination system for chromosome engineering in Escherichia coli. Proc Natl Acad Sci U S A 97: 5978–5983. 10811905 10.1073/pnas.100127597 PMC18544

[17] Szczepanska AK (2009) Bacteriophage-encoded functions engaged in initiation of homologous recombination events. Crit Rev Microbiol 35: 197–220. 19563302 10.1080/10408410902983129

[18] Lo Piano A, Martinez-Jimenez MI, Zecchi L, Ayora S (2011) Recombination-dependent concatemeric viral DNA replication. Virus Res 160: 1–14. 21708194 10.1016/j.virusres.2011.06.009

[19] Kuzminov A (1999) Recombinational repair of DNA damage in Escherichia coli and bacteriophage lambda. Microbiol Mol Biol Rev 63: 751–813 table of contents. 10585965 10.1128/mmbr.63.4.751-813.1999 PMC98976

[20] Wilkinson DE, Weller SK (2003) The role of DNA recombination in herpes simplex virus DNA replication. IUBMB Life 55: 451–458. 14609200 10.1080/15216540310001612237

[21] Kass EM, Jasin M (2010) Collaboration and competition between DNA double-strand break repair pathways. FEBS Lett 584: 3703–3708. 20691183 10.1016/j.febslet.2010.07.057 PMC3954739

[22] Wyman C, Kanaar R (2006) DNA double-strand break repair: all's well that ends well. Annu Rev Genet 40: 363–383. 16895466 10.1146/annurev.genet.40.110405.090451

[23] Iyer LM, Koonin EV, Aravind L (2002) Classification and evolutionary history of the single-strand annealing proteins, RecT, Redbeta, ERF and RAD52. BMC Genomics 3: 8. 11914131 10.1186/1471-2164-3-8 PMC101383

[24] Kawabata M, Kawabata T, Nishibori M (2005) Role of recA/RAD51 family proteins in mammals. Acta Med Okayama 59: 1–9. 15902993 10.18926/AMO/31987

[25] Singleton MR, Wentzell LM, Liu Y, West SC, Wigley DB (2002) Structure of the single-strand annealing domain of human RAD52 protein. Proc Natl Acad Sci U S A 99: 13492–13497. 12370410 10.1073/pnas.212449899 PMC129701

[26] Stark JM, Pierce AJ, Oh J, Pastink A, Jasin M (2004) Genetic steps of mammalian homologous repair with distinct mutagenic consequences. Mol Cell Biol 24: 9305–9316. 15485900 10.1128/MCB.24.21.9305-9316.2004 PMC522275

[27] Branzei D, Foiani M (2008) Regulation of DNA repair throughout the cell cycle. Nat Rev Mol Cell Biol 9: 297–308. 18285803 10.1038/nrm2351

[28] Shrivastav M, De Haro LP, Nickoloff JA (2008) Regulation of DNA double-strand break repair pathway choice. Cell Res 18: 134–147. 18157161 10.1038/cr.2007.111

[29] Harper JW, Elledge SJ (2007) The DNA damage response: ten years after. Mol Cell 28: 739–745. 18082599 10.1016/j.molcel.2007.11.015

[30] Lavin MF, Kozlov S (2007) DNA damage-induced signalling in ataxia-telangiectasia and related syndromes. Radiother Oncol 83: 231–237. 17512070 10.1016/j.radonc.2007.04.032

[31] DeFazio LG, Stansel RM, Griffith JD, Chu G (2002) Synapsis of DNA ends by DNA-dependent protein kinase. EMBO J 21: 3192–3200. 12065431 10.1093/emboj/cdf299 PMC126055

[32] Spagnolo L, Rivera-Calzada A, Pearl LH, Llorca O (2006) Three-dimensional structure of the human DNA-PKcs/Ku70/Ku80 complex assembled on DNA and its implications for DNA DSB repair. Mol Cell 22: 511–519. 16713581 10.1016/j.molcel.2006.04.013

[33] Valerie K, Povirk LF (2003) Regulation and mechanisms of mammalian double-strand break repair. Oncogene 22: 5792–5812. 12947387 10.1038/sj.onc.1206679

[34] Mohni KN, Livingston CM, Cortez D, Weller SK (2010) ATR and ATRIP are recruited to herpes simplex virus type 1 replication compartments even though ATR signaling is disabled. J Virol 84: 12152–12164. 20861269 10.1128/JVI.01643-10 PMC2976399

[35] Mohni KN, Mastrocola AS, Bai P, Weller SK, Heinen CD (2011) DNA mismatch repair proteins are required for efficient herpes simplex virus 1 replication. J Virol 85: 12241–12253. 21957315 10.1128/JVI.05487-11 PMC3209375

[36] Gregory DA, Bachenheimer SL (2008) Characterization of mre11 loss following HSV-1 infection. Virology 373: 124–136. 18177684 10.1016/j.virol.2007.12.005 PMC2295170

[37] Lees-Miller SP, Long MC, Kilvert MA, Lam V, Rice SA, et al (1996) Attenuation of DNA-dependent protein kinase activity and its catalytic subunit by the herpes simplex virus type 1 transactivator ICP0. J Virol 70: 7471–7477. 8892865 10.1128/jvi.70.11.7471-7477.1996 PMC190814

[38] Lilley CE, Carson CT, Muotri AR, Gage FH, Weitzman MD (2005) DNA repair proteins affect the lifecycle of herpes simplex virus 1. Proc Natl Acad Sci U S A 102: 5844–5849. 15824307 10.1073/pnas.0501916102 PMC556126

[39] Taylor TJ, Knipe DM (2004) Proteomics of herpes simplex virus replication compartments: association of cellular DNA replication, repair, recombination, and chromatin remodeling proteins with ICP8. J Virol 78: 5856–5866. 15140983 10.1128/JVI.78.11.5856-5866.2004 PMC415816

[40] Wilcock D, Lane DP (1991) Localization of p53, retinoblastoma and host replication proteins at sites of viral replication in herpes-infected cells. Nature 349: 429–431. 1671528 10.1038/349429a0

[41] Wilkinson DE, Weller SK (2004) Recruitment of cellular recombination and repair proteins to sites of herpes simplex virus type 1 DNA replication is dependent on the composition of viral proteins within prereplicative sites and correlates with the induction of the DNA damage response. J Virol 78: 4783–4796. 15078960 10.1128/JVI.78.9.4783-4796.2004 PMC387708

[42] Lilley CE, Chaurushiya MS, Boutell C, Everett RD, Weitzman MD (2011) The intrinsic antiviral defense to incoming HSV-1 genomes includes specific DNA repair proteins and is counteracted by the viral protein ICP0. PLoS Pathog 7: e1002084. 21698222 10.1371/journal.ppat.1002084 PMC3116817

[43] Lilley CE, Chaurushiya MS, Boutell C, Landry S, Suh J, et al (2010) A viral E3 ligase targets RNF8 and RNF168 to control histone ubiquitination and DNA damage responses. EMBO J 29: 943–955. 20075863 10.1038/emboj.2009.400 PMC2837166

[44] Parkinson J, Lees-Miller SP, Everett RD (1999) Herpes simplex virus type 1 immediate-early protein vmw110 induces the proteasome-dependent degradation of the catalytic subunit of DNA-dependent protein kinase. J Virol 73: 650–657. 9847370 10.1128/jvi.73.1.650-657.1999 PMC103871

[45] Shirata N, Kudoh A, Daikoku T, Tatsumi Y, Fujita M, et al (2005) Activation of ataxia telangiectasia-mutated DNA damage checkpoint signal transduction elicited by herpes simplex virus infection. J Biol Chem 280: 30336–30341. 15964848 10.1074/jbc.M500976200

[46] Muylaert I, Elias P (2007) Knockdown of DNA ligase IV/XRCC4 by RNA interference inhibits herpes simplex virus type I DNA replication. J Biol Chem 282: 10865–10872. 17296606 10.1074/jbc.M611834200

[47] Pierce AJ, Johnson RD, Thompson LH, Jasin M (1999) XRCC3 promotes homology-directed repair of DNA damage in mammalian cells. Genes Dev 13: 2633–2638. 10541549 10.1101/gad.13.20.2633 PMC317094

[48] Bennardo N, Cheng A, Huang N, Stark JM (2008) Alternative-NHEJ is a mechanistically distinct pathway of mammalian chromosome break repair. PLoS Genet 4: e1000110. 18584027 10.1371/journal.pgen.1000110 PMC2430616

[49] Severini A, Morgan AR, Tovell DR, Tyrrell DL (1994) Study of the structure of replicative intermediates of HSV-1 DNA by pulsed-field gel electrophoresis. Virology 200: 428–435. 8178432 10.1006/viro.1994.1206

[50] Goldstein DJ, Weller SK (1988) lacZ insertional mutagen is used to demonstrate that the UL52 gene of herpes simplex virus type 1 is required for virus growth and DNA synthesis. J Virol 62: 2970–2977. 2839713 10.1128/jvi.62.8.2970-2977.1988 PMC253735

[51] Lamberti C, Weller SK (1998) The herpes simplex virus type 1 cleavage/packaging protein, UL32, is involved in efficient localization of capsids to replication compartments. J Virol 72: 2463–2473. 9499108 10.1128/jvi.72.3.2463-2473.1998 PMC109547

[52] Lim SI, Min BE, Jung GY (2008) Lagging strand-biased initiation of red recombination by linear double-stranded DNAs. J Mol Biol 384: 1098–1105. 18983848 10.1016/j.jmb.2008.10.047

[53] Poteete AR (2008) Involvement of DNA replication in phage lambda Red-mediated homologous recombination. Mol Microbiol 68: 66–74. 18333884 10.1111/j.1365-2958.2008.06133.x

[54] Stahl FW, McMilin KD, Stahl MM, Crasemann JM, Lam S (1974) The distribution of crossovers along unreplicated lambda bacteriophage chromosomes. Genetics 77: 395–408. 4416166 10.1093/genetics/77.3.395 PMC1213136

[55] Li Z, Karakousis G, Chiu SK, Reddy G, Radding CM (1998) The beta protein of phage lambda promotes strand exchange. J Mol Biol 276: 733–744. 9500923 10.1006/jmbi.1997.1572

[56] Muniyappa K, Radding CM (1986) The homologous recombination system of phage lambda. Pairing activities of beta protein. J Biol Chem 261: 7472–7478. 2940241

[57] Stahl MM, Thomason L, Poteete AR, Tarkowski T, Kuzminov A, et al (1997) Annealing vs. invasion in phage lambda recombination. Genetics 147: 961–977. 9383045 10.1093/genetics/147.3.961 PMC1208271

[58] Gao M, Knipe DM (1989) Genetic evidence for multiple nuclear functions of the herpes simplex virus ICP8 DNA-binding protein. J Virol 63: 5258–5267. 2555553 10.1128/jvi.63.12.5258-5267.1989 PMC251191

[59] Weller SK, Seghatoleslami MR, Shao L, Rowse D, Carmichael EP (1990) The herpes simplex virus type 1 alkaline nuclease is not essential for viral DNA synthesis: isolation and characterization of a lacZ insertion mutant. J Gen Virol 71 (Pt 12) 2941–2952. 2177086 10.1099/0022-1317-71-12-2941

[60] Nimonkar AV, Boehmer PE (2002) In vitro strand exchange promoted by the herpes simplex virus type-1 single strand DNA-binding protein (ICP8) and DNA helicase-primase. J Biol Chem 277: 15182–15189. 11832483 10.1074/jbc.M109988200

[61] Nimonkar AV, Boehmer PE (2003) On the mechanism of strand assimilation by the herpes simplex virus type-1 single-strand DNA-binding protein (ICP8). Nucleic Acids Res 31: 5275–5281. 12954763 10.1093/nar/gkg740 PMC203323

[62] Balasubramanian N, Bai P, Buchek G, Korza G, Weller SK (2010) Physical interaction between the herpes simplex virus type 1 exonuclease, UL12, and the DNA double-strand break-sensing MRN complex. J Virol 84: 12504–12514. 20943970 10.1128/JVI.01506-10 PMC3004347

[63] Thomas MS, Gao M, Knipe DM, Powell KL (1992) Association between the herpes simplex virus major DNA-binding protein and alkaline nuclease. J Virol 66: 1152–1161. 1309895 10.1128/jvi.66.2.1152-1161.1992 PMC240819

[64] Antrobus R, Grant K, Gangadharan B, Chittenden D, Everett RD, et al (2009) Proteomic analysis of cells in the early stages of herpes simplex virus type-1 infection reveals widespread changes in the host cell proteome. Proteomics 9: 3913–3927. 19670248 10.1002/pmic.200900207

[65] Stark JM, Hu P, Pierce AJ, Moynahan ME, Ellis N, et al (2002) ATP hydrolysis by mammalian RAD51 has a key role during homology-directed DNA repair. J Biol Chem 277: 20185–20194. 11923292 10.1074/jbc.M112132200

[66] Goldstein JN, Weller SK (1998) The exonuclease activity of HSV-1 UL12 is required for in vivo function. Virology 244: 442–457. 9601512 10.1006/viro.1998.9129

[67] Shirasawa S, Furuse M, Yokoyama N, Sasazuki T (1993) Altered growth of human colon cancer cell lines disrupted at activated Ki-ras. Science 260: 85–88. 8465203 10.1126/science.8465203

[68] Koi M, Umar A, Chauhan DP, Cherian SP, Carethers JM, et al (1994) Human chromosome 3 corrects mismatch repair deficiency and microsatellite instability and reduces N-methyl-N′-nitro-N-nitrosoguanidine tolerance in colon tumor cells with homozygous hMLH1 mutation. Cancer Res 54: 4308–4312. 8044777

[69] Gunn A, Bennardo N, Cheng A, Stark JM (2011) Correct End Use during End Joining of Multiple Chromosomal Double Strand Breaks Is Influenced by Repair Protein RAD50, DNA-dependent Protein Kinase DNA-PKcs, and Transcription Context. J Biol Chem 286: 42470–42482. 22027841 10.1074/jbc.M111.309252 PMC3234933

[70] Brooks K, Clark AJ (1967) Behavior of lambda bacteriophage in a recombination deficienct strain of Escherichia coli. J Virol 1: 283–293. 4918235 10.1128/jvi.1.2.283-293.1967 PMC375226

[71] Echolas H, Gingery R (1968) Mutants of bacteriophage lambda defective in vegetative genetic recombination. J Mol Biol 34: 239–249. 4938547 10.1016/0022-2836(68)90249-0

[72] Franklin NC (1967) Deletions and functions of the center of the phi80 -lambda phage genome. Evidence for a phage function promoting genetic recombination. Genetics 57: 301–318. 5584569 10.1093/genetics/57.2.301 PMC1211728

[73] Shulman MJ, Hallick LM, Echols H, Signer ER (1970) Properties of recombination-deficient mutants of bacteriophage lambda. J Mol Biol 52: 501–520. 4923748 10.1016/0022-2836(70)90416-x

[74] Signer ER, Weil J (1968) Recombination in bacteriophage lambda. I. Mutants deficient in general recombination. J Mol Biol 34: 261–271. 5760458 10.1016/0022-2836(68)90251-9

[75] van de Putte P, Zwenk H, Rorsch A (1966) Properties of four mutants of Escherichia coli defective in genetic recombination. Mutat Res 3: 381–392. 5339653 10.1016/0027-5107(66)90048-0

[76] Kulkarni AS, Fortunato EA (2011) Stimulation of homology-directed repair at I-SceI-induced DNA breaks during the permissive life cycle of human cytomegalovirus. J Virol 85: 6049–6054. 21490102 10.1128/JVI.02514-10 PMC3126324

[77] Mimitou EP, Symington LS (2009) DNA end resection: many nucleases make light work. DNA Repair (Amst) 8: 983–995. 19473888 10.1016/j.dnarep.2009.04.017 PMC2760233

[78] Nimonkar AV, Genschel J, Kinoshita E, Polaczek P, Campbell JL, et al (2011) BLM-DNA2-RPA-MRN and EXO1-BLM-RPA-MRN constitute two DNA end resection machineries for human DNA break repair. Genes Dev 25: 350–362. 21325134 10.1101/gad.2003811 PMC3042158

[79] Sartori AA, Lukas C, Coates J, Mistrik M, Fu S, et al (2007) Human CtIP promotes DNA end resection. Nature 450: 509–514. 17965729 10.1038/nature06337 PMC2409435

[80] de Wind N, Dekker M, Berns A, Radman M, te Riele H (1995) Inactivation of the mouse Msh2 gene results in mismatch repair deficiency, methylation tolerance, hyperrecombination, and predisposition to cancer. Cell 82: 321–330. 7628020 10.1016/0092-8674(95)90319-4

[81] Lyndaker AM, Alani E (2009) A tale of tails: insights into the coordination of 3′ end processing during homologous recombination. Bioessays 31: 315–321. 19260026 10.1002/bies.200800195 PMC2958051

[82] Sugawara N, Paques F, Colaiacovo M, Haber JE (1997) Role of Saccharomyces cerevisiae Msh2 and Msh3 repair proteins in double-strand break-induced recombination. Proc Natl Acad Sci U S A 94: 9214–9219. 9256462 10.1073/pnas.94.17.9214 PMC23120

[83] Jacob RJ, Roizman B (1977) Anatomy of herpes simplex virus DNA VIII. Properties of the replicating DNA. J Virol 23: 394–411. 196115 10.1128/jvi.23.2.394-411.1977 PMC515842

[84] RW HY, Oakes JE, Kudler L (1977) In vitro repair of the preexisting nicks and gaps in herpes simplex virus DNA. Virology 76: 286–294. 189493 10.1016/0042-6822(77)90303-8

[85] Bataille D, Epstein A (1994) Herpes simplex virus replicative concatemers contain L components in inverted orientation. Virology 203: 384–388. 8053162 10.1006/viro.1994.1498

[86] Bataille D, Epstein AL (1997) Equimolar generation of the four possible arrangements of adjacent L components in herpes simplex virus type 1 replicative intermediates. J Virol 71: 7736–7743. 9311858 10.1128/jvi.71.10.7736-7743.1997 PMC192125

[87] Sarisky RT, Weber PC (1994) Requirement for double-strand breaks but not for specific DNA sequences in herpes simplex virus type 1 genome isomerization events. J Virol 68: 34–47. 8254746 10.1128/jvi.68.1.34-47.1994 PMC236261

[88] Rybalchenko N, Golub EI, Bi B, Radding CM (2004) Strand invasion promoted by recombination protein beta of coliphage lambda. Proc Natl Acad Sci U S A 101: 17056–17060. 15574500 10.1073/pnas.0408046101 PMC535401

[89] Maresca M, Erler A, Fu J, Friedrich A, Zhang Y, et al (2010) Single-stranded heteroduplex intermediates in lambda Red homologous recombination. BMC Mol Biol 11: 54. 20670401 10.1186/1471-2199-11-54 PMC2918612

[90] Mosberg JA, Lajoie MJ, Church GM (2010) Lambda red recombineering in Escherichia coli occurs through a fully single-stranded intermediate. Genetics 186: 791–799. 20813883 10.1534/genetics.110.120782 PMC2975298

[91] Bujnicki JM, Rychlewski L (2001) The herpesvirus alkaline exonuclease belongs to the restriction endonuclease PD-(D/E)XK superfamily: insight from molecular modeling and phylogenetic analysis. Virus Genes 22: 219–230. 11324759 10.1023/a:1008131810233

[92] Datsenko KA, Wanner BL (2000) One-step inactivation of chromosomal genes in Escherichia coli K-12 using PCR products. Proc Natl Acad Sci U S A 97: 6640–6645. 10829079 10.1073/pnas.120163297 PMC18686

[93] Murphy KC, Campellone KG, Poteete AR (2000) PCR-mediated gene replacement in Escherichia coli. Gene 246: 321–330. 10767554 10.1016/s0378-1119(00)00071-8

[94] Muyrers JP, Zhang Y, Stewart AF (2001) Techniques: Recombinogenic engineering–new options for cloning and manipulating DNA. Trends Biochem Sci 26: 325–331. 11343926 10.1016/s0968-0004(00)01757-6

[95] Campbell A (1994) Comparative molecular biology of lambdoid phages. Annu Rev Microbiol 48: 193–222. 7826005 10.1146/annurev.mi.48.100194.001205

[96] Bowden R, Sakaoka H, Donnelly P, Ward R (2004) High recombination rate in herpes simplex virus type 1 natural populations suggests significant co-infection. Infect Genet Evol 4: 115–123. 15157629 10.1016/j.meegid.2004.01.009

[97] Muylkens B, Farnir F, Meurens F, Schynts F, Vanderplasschen A, et al (2009) Coinfection with two closely related alphaherpesviruses results in a highly diversified recombination mosaic displaying negative genetic interference. J Virol 83: 3127–3137. 19153224 10.1128/JVI.02474-08 PMC2655596

[98] Thiry E, Meurens F, Muylkens B, McVoy M, Gogev S, et al (2005) Recombination in alphaherpesviruses. Rev Med Virol 15: 89–103. 15546129 10.1002/rmv.451

[99] Norberg P, Kasubi MJ, Haarr L, Bergstrom T, Liljeqvist JA (2007) Divergence and recombination of clinical herpes simplex virus type 2 isolates. J Virol 81: 13158–13167. 17881457 10.1128/JVI.01310-07 PMC2169075

[100] Topaloglu O, Hurley PJ, Yildirim O, Civin CI, Bunz F (2005) Improved methods for the generation of human gene knockout and knockin cell lines. Nucleic Acids Res 33: e158. 16214806 10.1093/nar/gni160 PMC1255732

[101] Goldstein JN, Weller SK (1998) In vitro processing of herpes simplex virus type 1 DNA replication intermediates by the viral alkaline nuclease, UL12. J Virol 72: 8772–8781. 9765421 10.1128/jvi.72.11.8772-8781.1998 PMC110293

[102] Reuven NB, Antoku S, Weller SK (2004) The UL12.5 gene product of herpes simplex virus type 1 exhibits nuclease and strand exchange activities but does not localize to the nucleus. J Virol 78: 4599–4608. 15078942 10.1128/JVI.78.9.4599-4608.2004 PMC387724

[103] Richardson C, Moynahan ME, Jasin M (1998) Double-strand break repair by interchromosomal recombination: suppression of chromosomal translocations. Genes Dev 12: 3831–3842. 9869637 10.1101/gad.12.24.3831 PMC317271

[104] Fattah F, Lee EH, Weisensel N, Wang Y, Lichter N, et al (2010) Ku regulates the non-homologous end joining pathway choice of DNA double-strand break repair in human somatic cells. PLoS Genet 6: e1000855. 20195511 10.1371/journal.pgen.1000855 PMC2829059

[105] Heilbronn R, zur Hausen H (1989) A subset of herpes simplex virus replication genes induces DNA amplification within the host cell genome. J Virol 63: 3683–3692. 2547992 10.1128/jvi.63.9.3683-3692.1989 PMC250959

[106] Livingston CM, DeLuca NA, Wilkinson DE, Weller SK (2008) Oligomerization of ICP4 and rearrangement of heat shock proteins may be important for herpes simplex virus type 1 prereplicative site formation. J Virol 82: 6324–6336. 18434395 10.1128/JVI.00455-08 PMC2447070

